# Desigualdades regionais no acesso ao parto hospitalar no Estado do Rio de Janeiro, Brasil: redes de deslocamento, distância e tempo (2010-2019)

**DOI:** 10.1590/0102-311XPT064423

**Published:** 2024-05-20

**Authors:** Lucas Lopes Felipe, Priscila Costa Albuquerque, Juliana Freitas Lopes, Fabio Zicker, Bruna de Paula Fonseca

**Affiliations:** 1 Universidade Federal do Rio de Janeiro, Rio de Janeiro, Brasil.; 2 Centro de Desenvolvimento Tecnológico em Saúde, Fundação Oswaldo Cruz, Rio de Janeiro, Brasil.

**Keywords:** Parturition, Social Network Analysis, Transportation, Health Services, Health Management, Parto, Análise de Rede Social, Meios de Transporte, Serviços de Saúde, Gestão em Saúde, Parto, Análisis de Redes Sociales, Transportes, Servicios de Salud, Gestión en Salud

## Abstract

A dificuldade de acesso aos serviços de atenção ao parto está associada à mortalidade infantil e neonatal e à morbimortalidade materna. Neste estudo, dados do Sistema Único de Saúde (SUS) foram utilizados para mapear a evolução da acessibilidade geográfica ao parto hospitalar de risco habitual no Estado do Rio de Janeiro, Brasil, correspondentes a 418.243 internações nos biênios 2010-2011 e 2018-2019. Foram estimados os fluxos de deslocamento, as distâncias percorridas e o tempo de deslocamento intermunicipal entre o município de residência e de internação das gestantes. Houve um crescimento de 15,9% para 21,5% na proporção de gestantes que precisaram se deslocar. A distância percorrida aumentou de 24,6 para 26km, e o tempo de deslocamento de 76,4 para 96,1 minutos, com grande variação entre as Regiões de Saúde (RS). As gestantes residentes na RS Centro Sul se deslocaram mais frequentemente (37,4-48,9%), e as residentes nas RS Baía da Ilha Grande e Noroeste percorreram as maiores distâncias (90,9-132,1km) e levaram mais tempo para chegar ao hospital no último biênio (96-137 minutos). A identificação dos municípios que receberam gestantes de muitos outros municípios e daqueles que atenderam maior volume de gestantes (núcleos e polos de atração, respectivamente) refletiu a indisponibilidade e as disparidades no acesso aos serviços. As desigualdades regionais e a redução da acessibilidade alertam para a necessidade de adequar a oferta à demanda e de revisar a distribuição dos serviços de atenção ao parto no Rio de Janeiro. O estudo contribui para as pesquisas e o planejamento sobre o acesso a serviços de saúde materno-infantil, além de servir como referência para outros estados do país.

## Abstract (en)

Difficult access to birth care services is associated with infant and neonatal mortality and maternal morbidity and mortality. In this study, data from the Brazilian Unified National Health System (SUS) were used to map the evolution of geographic accessibility to hospital birth of usual risk in the state of Rio de Janeiro, Brazil, corresponding to 418,243 admissions in 2010-2011 and 2018-2019. Travel flows, distances traveled, and intermunicipal travel time between the pregnant women’s municipality and hospital location were estimated. An increase from 15.9% to 21.5% was observed in the number of pregnant women who needed to travel. The distance traveled increased from 24.6 to 26km, and the travel time from 76.4 to 96.1 minutes, with high variation between Health Regions (HR). Pregnant women living in HR Central-South traveled more frequently (37.4-48.9%), and those living in the HRs Baía da Ilha Grande and Northwest traveled the largest distances (90.9-132.1km) and took more time to get to the hospital in 2018-2019 (96-137 minutes). The identification of municipalities that received pregnant women from many other municipalities and municipalities that treated a higher number of pregnant women (hubs and attraction poles, respectively) reflected the unavailability and disparities in access to services. Regional inequalities and reduced accessibility highlight the need to adapt supply to demand and review the distribution of birth care services in the state of Rio de Janeiro. This study contributes to research and planning on access to maternal and child health services and can be used as a reference study for other states in the country.

## Resumen

La dificultad para acceder a los servicios de atención al parto está asociada con la mortalidad infantil y neonatal, y con la morbimortalidad materna. En este estudio, se utilizaron datos del Sistema Único de Salud (SUS) para mapear la evolución de la accesibilidad geográfica al parto hospitalario de riesgo habitual en el estado de Río de Janeiro, Brasil, correspondiente a 418.243 hospitalizaciones en los bienios 2010-2011 y 2018-2019. Se estimaron los flujos de desplazamiento, las distancias recorridas y el tiempo de desplazamiento intermunicipal entre el municipio de residencia y la hospitalización de las mujeres embarazadas. Hubo un aumento del 15,9% al 21,5% en la proporción de mujeres embarazadas que necesitaron desplazarse. La distancia recorrida aumentó de 24,6 a 26km y el tiempo de desplazamiento de 76,4 a 96,1 minutos, con gran variación entre las Regiones de Salud (RS). Las mujeres embarazadas residentes en la RS Centro Sul se desplazaron con mayor frecuencia (37,4-48,9%), y las residentes en las RS Baía da Ilha Grande y Noroeste recorrieron las mayores distancias (90,9-132,1km) y tardaron más en llegar al hospital en el últimos bienio (96-137 minutos). La identificación de los municipios que recibieron mujeres embarazadas de muchos otros municipios y de aquellos que atendieron a un mayor volumen de mujeres embarazadas (núcleos y polos de atracción, respectivamente) reflejó la indisponibilidad y las disparidades en el acceso a los servicios. Las desigualdades regionales y la reducida accesibilidad alertan sobre la necesidad de adaptar la oferta a la demanda, y de revisar la distribución de los servicios de atención al parto en el estado de Rio de Janeiro. El estudio contribuye a las investigaciones y a la planificación sobre el acceso a los servicios de salud materno-infantil, y puede servir como referencia para otros estados del país.

## Introdução

No Brasil, 98% dos partos ocorrem em ambiente hospitalar e 77% na rede do Sistema Único de Saúde (SUS) ^1^. O acesso oportuno à internação para o parto é fundamental para garantir a segurança e a qualidade do cuidado materno. Apesar disso, a desigualdade geográfica na distribuição e acesso a serviços de atenção ao parto aponta para vazios assistenciais no SUS que fazem com que grande parte das gestantes precise se deslocar de um município a outro para ser devidamente assistida ^2^.

De 2011 a 2018, 99,2% dos partos realizados no Estado do Rio de Janeiro foram feitos no ambiente hospitalar ^3^. O Plano Diretor de Regionalização (PDR), estabelecido em 2001 e atualizado em 2012, definiu a organização e a execução de ações e serviços de saúde nos 92 municípios do estado, agrupados em nove Regiões de Saúde (RS) ^4^. Essas regiões devem ter suficiência em atenção básica e de média complexidade, além de algumas ações de alta complexidade, incluindo procedimentos de parto ^4^.

A acessibilidade geográfica é um dos componentes da avaliação do acesso aos serviços de saúde e representa uma dimensão relevante nos estudos sobre equidade nos sistemas de saúde pública ^5^. Ela expressa a adequação da distribuição espacial dos serviços às necessidades dos usuários, considerando a distância, os meios de transporte e o tempo de deslocamento ^6^. Refere-se à facilidade com que os residentes de uma determinada área podem chegar aos serviços e/ou unidades de saúde, o que pode ser avaliado por diferentes abordagens ^7^. Neste estudo, a acessibilidade geográfica é expressa como a distância física e o tempo de deslocamento entre o estabelecimento de saúde e o local de residência da gestante ^8^.

O atraso na atenção obstétrica está associado a desfechos maternos adversos ^9^^,^^10^, e o deslocamento de grandes distâncias para o parto é relacionado a níveis elevados de mortalidade infantil ^11^ e neonatal ^9^, além de haver maior risco de morbimortalidade materna ^12^. Embora as estratégias, como a Rede Cegonha, lançada no Brasil em 2011 ^13^, enfatizem a garantia de acesso aos cuidados obstétricos, não há registros sobre as distâncias percorridas ou sobre o tempo de deslocamento em busca da internação e indicadores para monitorar a evolução da acessibilidade à internação para o parto. A análise da distância percorrida e do tempo de deslocamento entre a residência até o hospital da rede pública constitui, portanto, uma avaliação útil e sensível para conhecer a dificuldade das gestantes em acessar cuidados maternos e neonatais.

O objetivo deste estudo é avaliar a acessibilidade geográfica ao parto hospitalar de risco habitual realizado no SUS no Estado do Rio de Janeiro nos últimos dez anos. Dados do Sistema de Informações Hospitalares (SIH) do SUS foram analisados em dois biênios (2010-2011 e 2018-2019), incluindo o período desde a implantação da Rede Cegonha no estado. Foram analisados a evolução dos fluxos de deslocamento, a distância percorrida e o tempo de deslocamento das gestantes em todas as regiões de saúde do Estado do Rio de Janeiro. Também foram identificados núcleos e polos de atenção à saúde nos quais provavelmente há maior demanda. O intuito é gerar evidências que subsidiem a avaliação, o monitoramento e a gestão dos serviços de saúde materno-infantil, utilizando uma metodologia que tem potencial de abrangência nacional e aplicabilidade a outras áreas de saúde pública.

## Método

O estudo utilizou dados dos biênios 2010-2011 e 2018-2019 que caracterizam dois momentos da atenção à saúde materno-infantil delimitadas pela *Portaria nº 1.459/2011*^13^ do Ministério da Saúde, que instituiu a Rede Cegonha. Essa Portaria introduziu uma mudança importante na forma de compreensão e organização da atenção à saúde materno-infantil, por meio de um modelo de atenção voltado ao pré-natal, parto e nascimento, puerpério e sistema logístico, que inclui transporte sanitário e regulação ^13^. A validação da escolha dos biênios foi realizada por uma análise de tendência, considerando os dez anos de janela temporal, que evidenciou uma tendência linear ascendente na evolução do percentual de gestantes que se deslocaram para realizar parto hospitalar. O modelo de tendência teve um bom ajuste aos dados, permitindo recortar o biênio final para fazer uma avaliação da evolução ao longo do tempo ^14^ (Material Suplementar: https://cadernos.ensp.fiocruz.br/static//arquivo/suppl-e00064423_8608.pdf).

Foram incluídos os registros de internação para o parto de gestantes residentes nos 92 municípios do Estado do Rio de Janeiro, nas nove RS, a saber: Centro Sul, Metropolitana I, Metropolitana II, Noroeste, Norte, Serrana, Baía da Ilha Grande, Baixada Litorânea e Médio Paraíba.

### Extração, processamento e validação dos dados

Dados de internação para o parto de risco habitual foram extraídos do SIH, por meio das Autorizações de Internação Hospitalar (AIH). O SIH foi primariamente construído sob uma perspectiva administrativa, mas pode ser considerado uma fonte importante de informações de saúde da população brasileira, além de um relevante instrumento para orientar gestores nas tomadas de decisão relacionadas ao planejamento das ações de saúde ^15^. O sistema é atualizado mensalmente e inclui informações sobre o serviço prestado, município de residência do paciente, município onde foi realizado o procedimento, entre outras informações.

O SIH foi acessado por meio da interface disponível na Plataforma de Ciência de Dados aplicada à Saúde (PCDaS; https://pcdas.icict.fiocruz.br/) do Instituto de Comunicação e Informação Científica e Tecnológica em Saúde, Fundação Oswaldo Cruz (ICICT/Fiocruz). O banco de dados foi construído em linguagem Python, considerando as seguintes variáveis: (1) ano de internação: 2010, 2011 e 2018, 2019; (2) procedimento realizado: códigos 411010034 para “PARTO CESARIANO”; e 310010039 para “PARTO NORMAL”; (3) idade da gestante: entre 10 e 49 anos; (4) município de residência: municípios do Estado do Rio de Janeiro; (5) município de internação para parto. O conjunto de dados foi construído com informações das AIH (não das gestantes individuais) e, para a análise, a quantidade de AIH foi utilizada como *proxy* para o número de gestantes. É importante ressaltar que apenas partos com risco habitual foram avaliados no intuito de evitar vieses relacionados a deslocamentos para a realização de partos de alto risco, uma vez que são direcionados para estabelecimentos específicos, mais complexos, com infraestrutura e equipes diferenciadas.

A variável cobrança de parto (motivo de saída/permanência) ^15^ foi utilizada para identificar os motivos de encerramento da internação para analisar possíveis associações entre os deslocamentos e desfechos de parto adversos (com morte materno-infantil). Os dados foram filtrados pelos códigos relacionados às cobranças por procedimento parto com desfecho adverso: alta da mãe/puérpera e óbito do recém-nascido (6.3); alta da mãe/puérpera com óbito fetal (6.4); óbito da gestante e do concepto (6.5); óbito da mãe/puérpera e alta do recém-nascido (6.6); e óbito da mãe/puérpera e permanência do recém-nascido (6.7). Três outros grupos de alta hospitalar também foram incluídos: certidão de óbito fornecida pelo médico assistente (4.1); certidão de óbito fornecida pelo Instituto Médico Legal (IML) (4.2); e certidão de óbito fornecida pelo Serviço de Verificação de Óbitos (SVO) (4.3).

Para analisar as possíveis associações entre os deslocamentos e indicadores socioeconômicos, foram utilizados dados agregados municipais e microdados do *Censo Demográfico 2010*, com o cruzamento das dimensões: situação socioeconômica e oferta e complexidade dos serviços de saúde para todas as RS do Rio de Janeiro, disponibilizados no banco de indicadores regionais e tipologia da plataforma Região e Redes ^16^, além dos dados do Índice de Desenvolvimento Humano Municipal (IDHM) dos 92 municípios do estado ^17^.

O estudo foi dispensado de revisão ética pelo Sistema de Comitês de Ética em Pesquisa da Comissão Nacional de Ética em Pesquisa (CEP/CONEP), por utilizar apenas dados públicos, do Departamento de Informática do SUS (DATASUS), sem identificação individual.

### Estimativa das distâncias percorridas e do tempo de deslocamento

As estimativas das distâncias percorridas e do tempo de deslocamento entre os municípios de residência (origem) e de internação para o parto (destino) foram obtidas por meio da interface de programação de aplicativos do Google (Distance Matrix Service; https://developers.google.com/maps/documentation/javascript/examples/distance-matrix), acessada com um *script* em Python 3.11 (https://docs.python.org/3.11/). A consulta foi realizada fixando os pares de deslocamento, apenas dos registros em que o município de residência e o município de internação eram diferentes. Procedimentos que envolviam deslocamentos interestaduais foram desconsiderados: 132 no período 2010-2011 e 327 no período 2018-2019, correspondendo a 0,1% e 0,2% do total de partos analisados em cada biênio, respectivamente.

As distâncias foram calculadas considerando o mapa rodoviário do Estado do Rio de Janeiro, a partir dos centroides de cada município. A análise do tempo foi realizada fixando a modalidade de deslocamento por transporte rodoviário (carro, *driving mode*) e transporte público (ônibus, *transit mode*) com base na variável *bestguess*, que é a melhor estimativa do tempo de deslocamento considerando médias históricas. Isso significa que as estimativas de tempo de deslocamento dos dois biênios analisados foram baseadas no mesmo conjunto histórico de dados, e não em dados individualizados para cada biênio. Para a estimativa do tempo de deslocamento no transporte rodoviário foi usada a variável *duration_in_traffic*, que inclui o tempo previsto no trânsito.

A informação sobre transporte público não foi igual para todos os municípios e RS, sendo a cobertura para Baía da Ilha Grande (100%), Metropolitana I (100%), Metropolitana II (100%), Baixada Litorânea (78%), Médio Paraíba (67%), Centro Sul (36%), Serrana (19%), Noroeste (15%) e Norte (11%). As análises regionais considerando a estimativa de tempo de deslocamento em transporte público foram feitas apenas para as RS que tinham dados disponíveis de pelo menos 50% de seus municípios. A distância e o tempo médios foram ponderados pelo número de gestantes que se deslocou entre um par de municípios origem/destino.

### Construção, visualização e análise das redes de deslocamento

Na rede, um par de municípios origem/destino define uma ligação e o número de gestantes que se deslocaram entre eles estabelece um fluxo. As ligações entre os municípios foram direcionais (da origem ao destino), assimétricas (não recíprocas) e ponderadas pelo número de gestantes que se deslocaram entre cada par de municípios. Os fluxos de deslocamento foram quantificados pelo número total de ligações entre os municípios e pela densidade das redes (número de ligações existentes em relação ao número de ligações possíveis). A visualização das redes e cálculo de métricas foram feitas no Gephi 0.9.10 (https://gephi.org/).

Com base na análise de redes sociais, foram caracterizados municípios “núcleo” e municípios “polo de atração” para atenção ao parto usando as seguintes métricas: (1) núcleo é identificado pela centralidade de grau de entrada, que quantifica o número total de conexões únicas em direção ao município de internação, ou seja, reflete o número de diferentes municípios de onde as gestantes se deslocaram até o local em que foi realizado o parto; (2) polo de atração é identificado pela centralidade de grau de entrada ponderada, que considera o número total de ligações únicas e o número de gestantes que se deslocaram em direção ao município de internação, ou seja, apresenta a quantidade de gestantes que se deslocaram até aquele município.

### Análise estatística

A análise estatística foi realizada com o Minitab v.20 (http://www.minitab.com). Os dados foram analisados quanto à distribuição normal, e o teste t-Student foi aplicado para comparar subgrupos em relação às médias ponderadas das distâncias percorridas e do tempo de deslocamento nos dois períodos. Foi levado em consideração um nível de significância estatística de p = 0,05.

## Resultados

**Figura 1:**
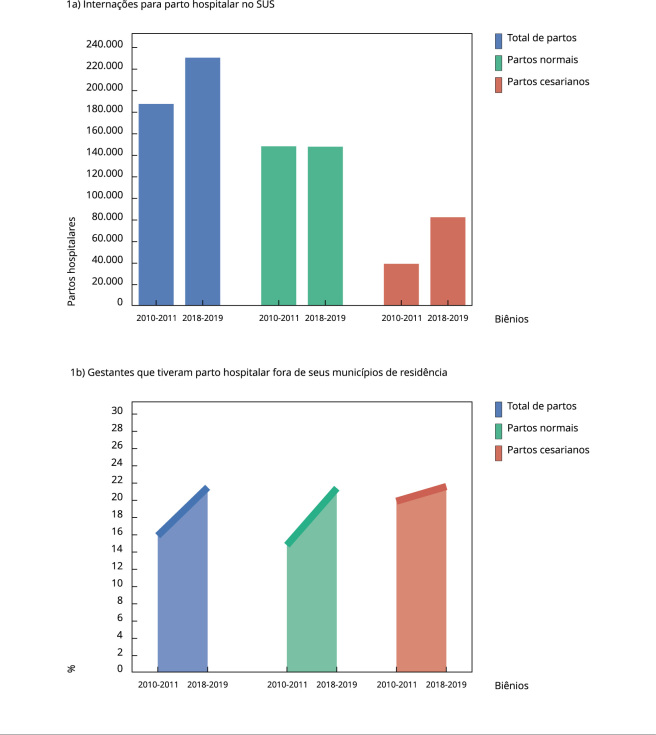
Número total de internações para parto hospitalar no Sistema Único de Saude (SUS) e porcentagem de gestantes que tiveram parto hospitalar fora de seus municípios de residência. Estado do Rio de Janeiro, Brasil, 2010-2011 e 2018-2019.

Foram analisados 418.243 partos: 296.337 normais (70,9%) e 121.906 cesarianos (29,1%). O número de partos normais permaneceu estável entre os dois biênios, com 148.327 e 148.010 procedimentos, enquanto o número de partos cesarianos duplicou no mesmo período: de 39.323 para 82.583 procedimentos (Figura 1a).

Nos dois biênios, 79.279 gestantes (18,9%) se deslocaram de seus municípios de residência para se internar para o parto (Figura 1b). Proporcionalmente, esse deslocamento se deu em maior grau nos partos cesarianos (21,1%) quando comparados aos partos normais (18,1%) (Figura 1b). Ao longo do tempo, o deslocamento intermunicipal em busca de internação para o parto aumentou de 15,9% para 21,5% (Figura 1b), sendo de 14,8% para 21,4% nos partos normais, e de 19,9% para 21,7% nos partos cesarianos (Figura 1b).

**Figura 2:**
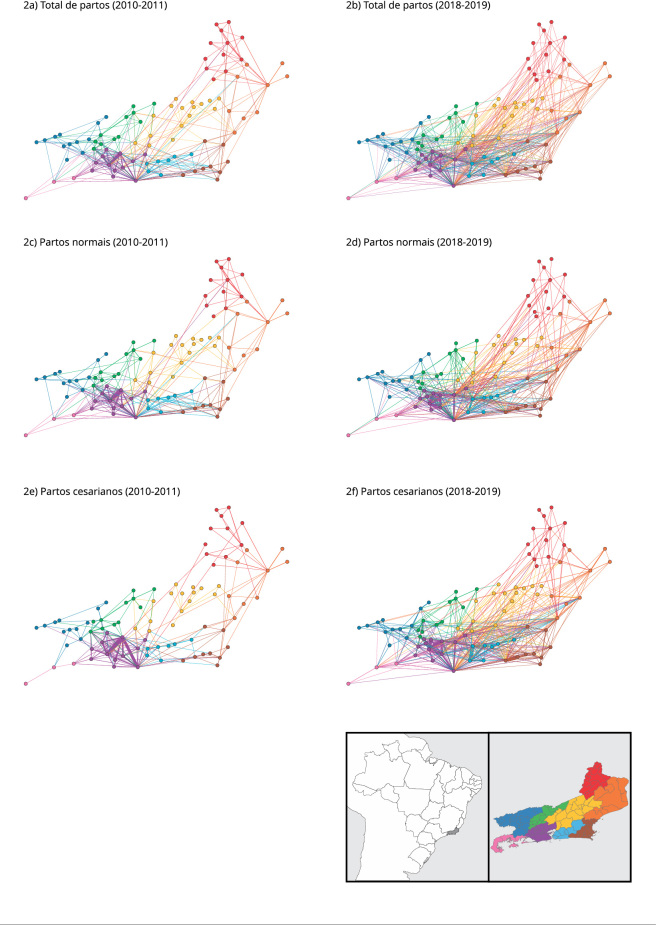
Redes de deslocamento intermunicipal para parto hospitalar no Sistema Único de Saúde (SUS) no Estado do Rio de Janeiro, Brasil, 2010-2011 e 2018-2019.

As redes de deslocamento das gestantes são apresentadas na Figura 2. Cada círculo representa um município e as ligações entre eles indicam o fluxo de origem/destino de gestantes. A cor da ligação indica a RS de residência da gestante. O fluxo de deslocamento intermunicipal para parto no período aumentou 86,3%. Esse aumento foi mais expressivo para os partos cesarianos (128,9%), comparados aos partos normais (46,3%). A densidade das redes de deslocamento também aumentou, evidenciando maior fluxo de gestantes entre municípios (Tabela 1).

Nota: cada círculo representa um município e as ligações entre eles indicam um fluxo de origem/destino de gestantes. A cor dos círculos e das ligações indica a Região de Saúde em que a gestante residia: Centro Sul (verde), Metropolitana I (roxo), Metropolitana II (azul claro), Noroeste (vermelho), Norte (laranja), Serrana (amarelo), Baía da Ilha Grande (rosa), Baixada Litorânea (marrom), Médio Paraíba (azul escuro).

**Tabela 1:** Métricas da rede de deslocamento de gestantes residentes no Estado do Rio de Janeiro, Brasil.

Tipo de parto	2010-2011			2018-2019		
	Nós	Ligações	Densidade da rede	Nós	Ligações	Densidade da rede
Normal	91	393	0,048	91	575	0,070
Cesariano	89	284	0,036	92	650	0,078
Total	92	453	0,054	92	844	0,101

A maioria dos deslocamentos respeitou os limites das RS, com um discreto aumento entre os biênios do deslocamento entre RS: de 6,9% para 7,3%. Além dos fluxos observados em direção aos municípios das RS Metropolitana I e II, houve também fluxos entre os municípios das RS Norte (laranja) e Noroeste (vermelho), e entre Baía da Ilha Grande (rosa) e Médio Paraíba (azul escuro) (Figura 2).

A distância média ponderada de deslocamento para o parto aumentou 5,7% entre os biênios: de 24,6 para 26km. Não foram observadas diferenças marcantes nos partos normais (24,2 *vs.* 24,2km), contudo para os partos cesarianos a distância percorrida aumentou 13,6% (25,6 *vs.* 29,1km) (Tabela 2).

**Tabela 2:** Distâncias percorridas e tempo de deslocamento intermunicipal para o parto hospitalar no Sistema Único de Saúde (SUS) nos biênios 2010-2011 e 2018-2019. Estado do Rio de Janeiro, Brasil.

Tipo de parto	2010-2011			2018-2019		
	Gestantes *	Média (DP)	Média ponderada	Gestantes *	Média (DP)	Média ponderada
Distância percorrida (km)						
Normal	21.929	59,6 (50,4)	24,2	31.667	92,6 (71,4)	24,2
Cesariano	7.808	56,2 (50,1)	25,5	17.875	93,2 (73,8)	29,1
Total	29.737	58,2 (50,4)	24,6	49.542	92,9 (72,7)	26,0
Tempo de deslocamento (minutos) - transporte rodoviário (carro)						
Normal	21.929	68,2 (46,5)	37,3	31.667	97,4 (63,8)	39,9
Cesariano	7.808	65,7 (44,7)	39,1	17.875	98,2 (66,4)	42,0
Total	29.737	67,2 (45,8)	37,8	49.542	97,8 (65,2)	40,6
Tempo de deslocamento (minutos) - transporte público (ônibus)						
Normal	19.203	178,8 (133,6)	76,0	28.411	233,9 (159,7)	92,3
Cesariano	6.815	162,6 (137,6)	77,5	14.271	245,4 (168,5)	103,7
Total	26.018	171,9 (135,4)	76,4	42.682	239,9 (164,3)	96,1

DP: desvio padrão.

* O número de internações foi usado como *proxy* do número de gestantes.

Nos dois biênios, a maioria das gestantes que se deslocaram (61,9% em 2010-2011 e 59,2% em 2018-2019) percorreram até 25km para realizar o parto hospitalar no SUS (Tabela 3). O resultado foi semelhante para as gestantes que realizaram parto normal (63,1% *vs.* 63%) e houve uma pequena redução para as gestantes que realizaram parto cesariano (58,5% *vs.* 52,7%). Essa redução foi acompanhada por um aumento na proporção de gestantes que percorreram mais de 51km para realizar parto cesariano no último biênio (8,7% *vs.* 12%) (Tabela 3).

**Tabela 3:** Faixas de distâncias percorridas e tempo de deslocamento intermunicipal para o parto hospitalar no Sistema Único de Saúde (SUS) nos biênios 2010-2011 e 2018-2019. Estado do Rio de Janeiro, Brasil.

Tipo de parto	Gestantes [n (%)] *									
	2010/2011					2018-2019				
	Distância percorrida para parto hospitalar (km)									
	< 10	11-25	26-50	51-100	> 100	< 10	11-25	26-50	51-100	> 100
Normal	2.205 (10,1)	11.631 (53,0)	6.463 (29,5)	1.525 (7,0)	105 (0,5)	11.070 (35,0)	8.870 (28,0)	9.368 (29,6)	1.984 (6,3)	375 (1,2)
Cesariano	910 (11,7)	3.652 (46,8)	2.565 (32,9)	644 (8,2)	37 (0,5)	4.701 (26,3)	4.724 (26,4)	6.301 (35,3)	1.731 (9,7)	418 (2,3)
Total	3.115 (10,5)	15.283 (51,4)	9.028 (30,4)	2.169 (7,3)	142 (0,5)	15.771 (31,8)	13.594 (27,4)	15.669 (31,6)	3.715 (7,5)	793 (1,6)
	Tempo de deslocamento - transporte rodoviário (carro)									
	< 15 minutos	16-30 minutos	31 minutos-1 hora	1-2 horas	> 2 horas	< 15 minutos	16-30 minutos	31 minutos-1 hora	1-2 horas	> 2 horas
Normal	134 (0,6)	9.064 (41,3)	11.107 (50,6)	1.547 (7,1)	77 (0,4)	172 (0,5)	7.588 (24,0)	20.617 (65,1)	2.938 (9,3)	352 (1,1)
Cesariano	48 (0,6)	2.810 (36,0)	4.248 (54,4)	672 (8,6)	30 (0,4)	425 (2,4)	5.129 (28,7)	9.952 (55,7)	1.984 (11,1)	385 (2,2)
Total	182 (0,6)	11.874 (39,9)	15.355 (51,6)	2.219 (7,5)	107 (0,4)	597 (1,2)	12.717 (25,7)	30.569 (61,7)	4.922 (9,9)	737 (1,5)
	Tempo de deslocamento - transporte público (ônibus)									
	< 15 minutos	16-30 minutos	31 minutos-1 hora	1-2 horas	> 2 horas	< 15 minutos	16-30 minutos	31 minutos-1 hora	1-2 horas	> 2 horas
Normal	0 (0,0)	938 (4,9)	9.123 (47,5)	6.459 (33,6)	2.683 (14,0)	0 (0,0)	0 (0,0)	6.123 (21,6)	17.771 (62,5)	4.517 (15,9)
Cesariano	0 (0,0)	380 (5,6)	2.638 (38,7)	2.991 (43,9)	806 (11,8)	0 (0,0)	0 (0,0)	2.762 (19,4)	8.429 (59,1)	3.080 (21,6)
Total	0 (0,0)	1.318 (5,1)	11.761 (45,2)	9.450 (36,3)	3.489 (13,4)	0 (0,0)	0 (0,0)	8.885 (20,8)	26.200 (61,4)	7.597 (17,8)

* O número de internações foi usado como *proxy* do número de gestantes. As porcentagens são relativas ao total de gestantes que se deslocaram de seus municípios para realizar parto hospitalar.

O tempo de deslocamento rodoviário (carro) até o hospital aumentou de 37,8 para 40,6 minutos entre os biênios. Utilizando o transporte público (ônibus), o tempo de deslocamento se eleva em 25,8% e aumenta ao longo dos anos: de 76,4 para 96,1 minutos. Não foram observadas diferenças marcantes entre as gestantes que realizaram partos normais e aquelas que realizaram partos cesarianos (Tabela 2). O tempo de deslocamento em transporte público aumentou entre os biênios. Enquanto 45,2% das gestantes levaram menos de 60 minutos para chegar ao hospital no primeiro biênio, 61,4% fizeram o percurso em tempo superior a 60 minutos na década seguinte (Tabela 3). O percurso ao hospital foi superior a 30 minutos para todas as gestantes em 2018-2019 (Tabela 3).

**Figura 3:**
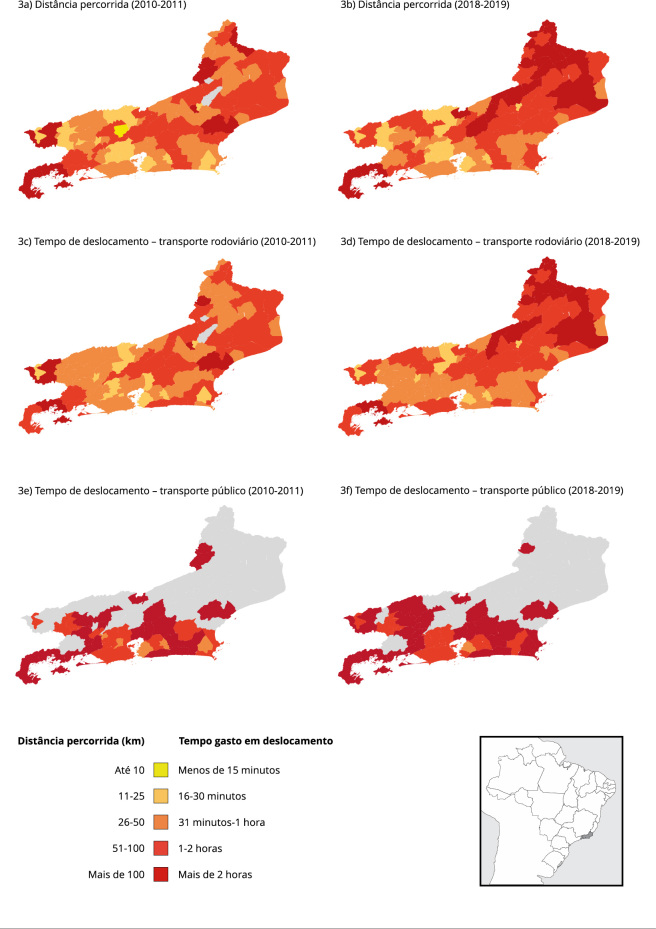
Representação da distância intermunicipal percorrida e tempo de deslocamento médios para realizar parto hospitalar no Sistema Único de Saúde (SUS), no Estado do Rio de Janeiro, Brasil.

A RS de residência influenciou nas distâncias e tempos de deslocamento até o hospital nos dois biênios analisados. Nas Figuras 3a e 3b, os municípios em cores mais escuras (principalmente nas RS Baía da Ilha Grande e Noroeste, e em alguns das RS Norte e Serrana), indicam distâncias maiores. Alguns dos municípios das RS Metropolitana I e II, Médio Paraíba e Centro Sul, em cores mais claras, foram aqueles cujas gestantes residentes percorreram as menores distâncias. O tempo de deslocamento em transporte rodoviário apresentou padrão semelhante (Figuras 3c e 3d). No transporte público, o tempo de deslocamento é superior para todas as RS analisadas, destacando municípios com percursos superiores a duas horas (Figuras 3e e 3f).

Nota: a média das distâncias e do tempo de deslocamento entre cada par de munícipios de origem/destino foi estimada e ponderada de acordo com o número de gestantes que viajaram entre eles. Mapas 3a e 3c: os municípios de cor cinza não registraram deslocamentos de gestantes no período analisado. Mapas 3e e 3f: os municípios de cor cinza não apresentavam dados disponíveis.

Não foram encontradas diferenças ou associações significativas, isoladamente ou agregadas, entre as distâncias percorridas e tempo de deslocamento em relação a desfechos adversos no parto ou indicadores socioeconômicos (dados não mostrados).

A Tabela 4 mostra que, ao longo do tempo, em sete das nove RS do Estado do Rio de Janeiro (Serrana, Norte, Noroeste, Metropolitana II, Médio Paraíba, Baixada Litorânea e Baía da Ilha Grande) houve aumento significativo da distância percorrida pelas gestantes. A RS Centro Sul foi a que apresentou a maior porcentagem de gestantes que se deslocaram (37,4% a 48,9%), e em oito dos 11 municípios todas as gestantes residentes se deslocaram para realizar o parto (dados não mostrados). A RS Baía da Ilha Grande foi a que apresentou a maior distância percorrida: 81,4km em 2010-2011 e 90,9km em 2018-2019. A RS Metropolitana II teve o maior aumento percentual de gestantes com deslocamento intermunicipal entre os períodos: de 7,6% para 27,4%. A RS Noroeste foi a única a mostrar uma redução no percentual de gestantes residentes que se deslocaram (de 22% para 5,5%); porém, apresentou o maior aumento da distância percorrida (121%) ao longo do tempo: de 59,8km para 132,1km. Somente a RS Metropolitana I apresentou diminuição significativa da distância percorrida (Tabela 4).

**Tabela 4:** Distâncias percorridas e tempo de deslocamento de gestantes para realizar parto hospitalar no Sistema Único de Saúde (SUS), segundo a Região de Saúde (RS) de residência no Estado do Rio de Janeiro, Brasil *.

RS	Distância percorrida (km)					Tempo de deslocamento (minutos) - transporte rodoviário (carro)					Tempo de deslocamento (minutos) - transporte público (ônibus)				
	2010-2011		2018-2019		Diferença (%) ***	2010-2011		2018-2019		Diferença (%) ***	2010-2011		2018-2019		Diferença (%) ***
	Gestantes [n (%)] **	Média ponderada	Gestantes [n (%)] **	Média ponderada		Gestantes [n (%)] **	Média ponderada	Gestantes [n (%)] **	Média ponderada		Gestantes [n (%)] **	Média ponderada	Gestantes [n (%)] **	Média ponderada	
Todas	29.737 (15,8)	24,6	49.542 (21,5)	26,0	6	29.737 (15,8)	37,8	49.542 (21,5)	40,6	7	26.018 (13,9)	76,4	42.682 (18,5)	96,1	26
Serrana	385 (4,2)	41,7	1.075 (8,4)	54,6	31	385 (4,2)	49,9	1.075 (8,4)	61,2	23	-	-	-	-	NA
Norte	997 (9,3)	46,9	1.447 (11,0)	54,7	17	997 (9,3)	56,1	1.447 (11,0)	60,9	9	-	-	-	-	NA
Noroeste	322 (22,0)	59,8	165 (5,5)	132,1	121	322 (22,0)	63,6	165 (5,5)	137,8	117	-	-	-	-	NA
Metropolitana I	23.130 (19,4)	22,6	32.222 (23,1)	21,6	-5	23.130 (19,4)	37,2	32.222 (23,1)	39,1	5	23.076 (19,4)	73,0	31.831 (22,8)	88,6	21
Metropolitana II	1.494 (7,6)	23,0	6.117 (27,4)	26,7	16	1.494 (7,6)	29,8	6.117 (27,4)	33,8	13	1.489 (7,5)	83,2	5.958 (26,7)	92,0	11
Médio Paraíba	517 (5,7)	27,6	1.034 (8,5)	30,2	10	517 (5,7)	38,2	1.034 (8,5)	39,2	3	272 (3,0)	142,7	517 (4,3)	129,6	-9
Centro Sul	1.852 (37,4)	21,4	3.016 (48,9)	21,0	-2	1.852 (37,4)	29,3	3.016 (48,9)	28,0	-4	-	-	-	-	NA
Baixada Litorânea	925 (9,9)	29,7	4.243 (27,2)	36,6	23	925 (9,9)	41,5	4.243 (27,2)	52,5	27	709 (7,6)	70,8	3.321 (21,3)	126,7	79
Baía da Ilha Grande	115 (3,1)	81,4	223 (4,3)	90,9	12	115 (3,1)	88,9	223 (4,3)	96,7	9	114 (3,0)	255,9	209 (4,0)	305,9	20

NA: não analisado.

Nota: valores em negrito, p < 0,05.

* Dados referentes às gestantes que se deslocaram de seus municípios para realizar parto hospitalar;

** Número de gestantes (em deslocamento): o número de internações fora do município de residência foi usado como *proxy* do número de gestantes. As porcentagens são relativas ao total de gestantes residentes na RS;

*** Diferença entre as médias ponderadas dos biênios.

O deslocamento por transporte rodoviário (carro) também mostrou aumento significativo em cinco das sete RS do estado (Serrana, Norte, Noroeste, Metropolitana I, Metropolitana II, e Baixada Litorânea) (Tabela 4). O tempo de deslocamento por carro na RS Baía da Ilha Grande foi o maior do estado e aumentou de 88,9 para 96,7 minutos ao longo do tempo. Em transporte público (ônibus), o tempo aumenta e passa de 255,9 para 305,9 minutos no período de estudo. A RS Noroeste foi a que apresentou maior aumento percentual no tempo de deslocamento por transporte rodoviário ao longo do tempo (117%), indo de 63,6 minutos para 137,8 minutos entre os dois biênios. A RS Baixada Litorânea apresentou o maior aumento no tempo de deslocamento por transporte público (79%): de 70,8 minutos para 126,7 minutos. As RS Serrana, Norte, Noroeste e Centro Sul tiveram baixa cobertura dos dados de transporte público e não foram consideradas na análise.

**Figura 4:**
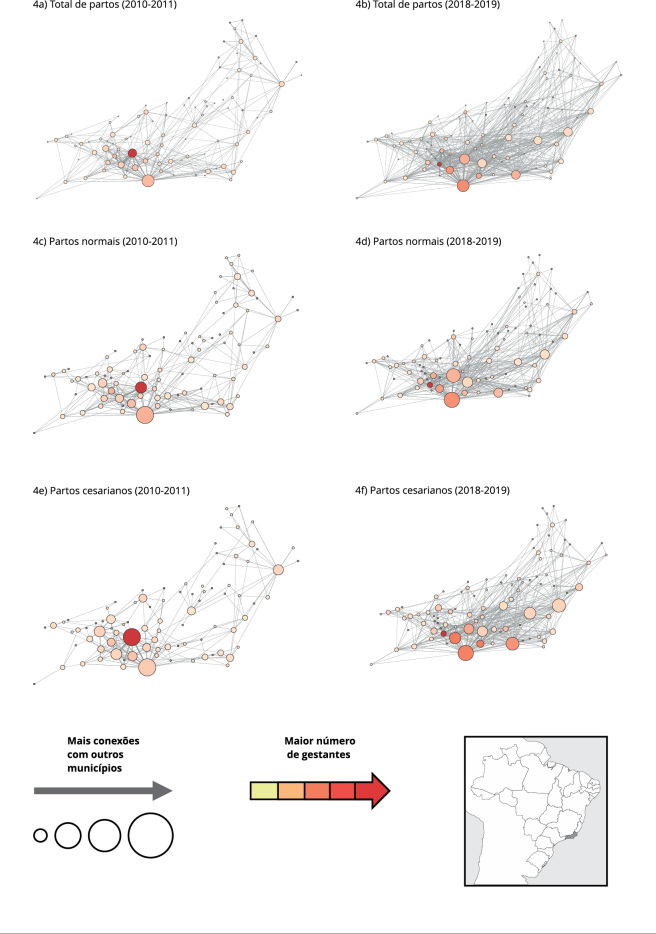
Municípios núcleos e municípios polos de atração para atenção ao parto no Estado do Rio de Janeiro, Brasil.

A Figura 4 mostra as redes de deslocamento das gestantes destacando os municípios de destino. Cada círculo representa um município e o tamanho e cor são proporcionais ao número de conexões com outros municípios e ao número de gestantes recebidas, respectivamente. Municípios de destino com muitas conexões com outros municípios foram considerados núcleos de atenção ao parto, representados no mapa com maior diâmetro. Municípios que receberam maior número de gestantes oriundas de um ou vários municípios foram considerados polos de atração, representados no mapa com cores mais avermelhadas.

Nota: cada círculo representa um município e uma ligação entre eles indica um fluxo origem/destino de gestantes. O diâmetro dos círculos é proporcional ao número de ligações com diferentes municípios. A intensidade da cor é proporcional à quantidade de gestantes vindas de outros municípios.

A Tabela 5 mostra os principais núcleos e polos de atração para a atenção ao parto nos dois períodos. A maioria dos núcleos e polos estava localizada nas RS Metropolitana I e II. Os municípios do Rio de Janeiro, Belford Roxo e Nova Iguaçu foram os principais núcleos de atenção ao parto em 2010-2011, recebendo gestantes de 37, 25 e 17 municípios diferentes, respectivamente. No segundo biênio, o Rio de Janeiro permaneceu como principal núcleo, recebendo gestantes de 62 municípios diferentes (67,5% mais que no período anterior), e Duque de Caxias e Saquarema aparecem como novos núcleos de atenção ao parto, recebendo gestantes de 51 e 44 de outros municípios, respectivamente.

**Tabela 5:** Municípios núcleo e municípios polos de atração para atenção ao parto pelo Sistema Único de Saúde (SUS) no Estado do Rio de Janeiro, Brasil.

Biênio/Tipo de parto	Ranking	Núcleos			Polos de atração		
		Município de destino	Conexões com outros municípios	Municípios atendidos (%)	Município de destino	Conexões com outros municípios	Municípios atendidos (%)
2010-2011							
Normal	1	Rio de Janeiro	33	35,9	Belford Roxo	7.991	56,2
	2	Belford Roxo	20	21,7	Rio de Janeiro	1.971	3,8
	3	Paracambi	15	16,3	Queimados	1.839	52,2
Cesariano	1	Belford Roxo	22	23,9	Belford Roxo	3.460	69,0
	2	Rio de Janeiro	22	23,9	Queimados	564	47,0
	3	Nova Iguaçu	13	14,1	São João de Meriti	549	58,3
Total	1	Rio de Janeiro	37	40,2	Belford Roxo	11.451	59,5
	2	Belford Roxo	25	27,2	Rio de Janeiro	2.405	3,9
	3	Nova Iguaçu	17	18,5	Queimados	2.403	50,9
2018-2019							
Normal	1	Rio de Janeiro	53	57,6	Mesquita	9.605	87,1
	2	Duque de Caxias	47	51,1	Rio de Janeiro	3.981	6,6
	3	São Gonçalo	34	37,0	São João de Meriti	3.303	55,8
Cesariano	1	Rio de Janeiro	46	50,0	Mesquita	3.537	87,7
	2	Macaé	39	42,4	São João de Meriti	1.855	66,1
	3	Saquarema	38	41,3	Niterói	1.829	44,6
Total	1	Rio de Janeiro	62	67,4	Mesquita	13.142	87,3
	2	Duque de Caxias	51	55,4	Rio de Janeiro	5.721	6,9
	3	Saquarema	44	47,8	São João de Meriti	5.158	59,1

Considerando o volume de gestantes recebidas e os dois tipos de parto, em 2010-2011 os municípios de Belford Roxo, Rio de Janeiro e Queimados foram os principais polos de atração para a atenção ao parto, recebendo 11.451, 2.405 e 2.403 gestantes no período, respectivamente (Tabela 5). Em 2018-2019, Belford Roxo e Queimados não aparecem mais como polos importantes para parto hospitalar e os polos passam a incluir, além do Rio de Janeiro, os municípios de Mesquita e São João de Meriti, que receberam 13.142, 5.721 e 5.158 gestantes, respectivamente. Vale ressaltar que a porcentagem de gestantes não residentes que realizaram parto hospitalar, normal ou cesariano, em Mesquita chegou a 87% de todos os partos do município.

## Discussão

O planejamento da acessibilidade aos serviços hospitalares é um dos principais aspectos para alcançar a universalidade, equidade e a integralidade da atenção à saúde. Neste estudo, examinamos pela primeira vez a evolução da acessibilidade geográfica ao parto hospitalar no Estado do Rio de Janeiro, mapeando fluxos preferenciais de deslocamento, distâncias percorridas e tempo de deslocamento de gestantes para realizar parto no SUS.

Embora se tenha registro de aumento nas taxas de parto cesariano no Brasil na última década (de 52,4% em 2010 para 56,3% em 2019) ^3^, os registros extraídos por este estudo mostraram que, no Estado do Rio de Janeiro, esse percentual foi de 29%. Entretanto, o aumento na última década sugere que ainda há importantes desafios para que os partos cesarianos não excedam 15% do total de partos, como recomendado pela Organização Mundial da Saúde (OMS) ^18^.

No período estudado, aproximadamente 80 mil gestantes (18,9%) residentes no Estado do Rio de Janeiro saíram de seus municípios de residência para realizar parto hospitalar no SUS. Apesar de um maior percentual de gestantes terem se deslocado para realizar parto cesariano nos primeiros anos de análise, as gestantes que realizaram parto normal passaram, ao longo do tempo, a sair mais de seus municípios de residência em busca de internação para o parto. No Brasil, houve fortalecimento, com o passar dos anos, de um movimento de mulheres em busca de informações sobre gestação e parto, que culminaram em diferentes espaços de comunicação para divulgação, troca de informações e para a reunião daquelas que almejavam partos normais ^19^, incluindo maternidades em que essa prática era mais frequente. Considerando o alto índice de partos cesarianos praticados na rede privada do Estado do Rio de Janeiro (67,9%) ^20^, a busca pelo SUS para a realização de parto normal por gestantes com convênio médico pode ter influenciado esse aumento. Em 2018-2019, foram mais de 7 mil procedimentos de parto normal ressarcidos ao SUS pelas operadoras de planos de saúde ^21^. Adicionalmente, recursos médicos limitados (equipes multiprofissionais reduzidas e ausência de plantonistas) em alguns municípios do estado poderiam implicar na indicação de cesarianas agendadas para garantir cuidados adequados à mãe e ao bebê ^22^. Um estudo mais aprofundado das características e das disparidades sociodemográficas dos municípios que possam influenciar o deslocamento das gestantes poderia fornecer informações complementares e relevantes à análise.

O fato de a maioria dos fluxos de deslocamento das gestantes obedecerem aos limites das RS evidencia que a organização dos serviços de atenção ao parto atende às necessidades no âmbito regional. Um exemplo são as gestantes que residem em municípios menores ou com baixa taxa de natalidade, que são necessariamente atendidas em municípios próximos previamente contemplados no desenho da programação e do pacto entre os municípios para atendimento das gestantes e dos nascimentos. Entretanto, o acesso a esses serviços no Estado do Rio de Janeiro é desigual e não melhorou ao longo do tempo. A porcentagem de gestantes que se deslocaram, as distâncias percorridas e o tempo de deslocamento aumentaram nos últimos dez anos, e as RS apresentaram perfis muito diferentes.

O deslocamento para internação para o parto está inserido no modelo das “três demoras”, criado para avaliar o acesso a cuidados obstétricos de emergência ^23^. A demora relacionada à acessibilidade ao serviço, seja por distribuição inadequada de serviços de saúde, distância e tempo de deslocamento, ou disponibilidade e custo de transporte, enfatiza o fato de que o tempo na obtenção de cuidados adequados é um fator relacionado a fatalidades ^23^. Estudos anteriores, realizados na Região Metropolitana do Rio de Janeiro, já evidenciaram que a distância/tempo entre a residência e o hospital são determinantes importantes para a morte materna ^24^. Apesar de não termos encontrado associação da distância/tempo com desfechos adversos no parto, é importante produzir evidências empíricas para estabelecer padrões de distância e tempo que sejam razoáveis para a realização de parto hospitalar. A atual regulamentação do Ministério da Saúde estabelece que os serviços de atenção obstétrica sejam distribuídos na proporção de 0,28 leitos obstétricos por 1.000 habitantes dependentes do SUS, mas não menciona a distância ou as áreas de abrangência ^25^.

A Rede Cegonha implementou a vinculação da gestante a uma maternidade durante o pré-natal, reduzindo a sua peregrinação no momento do parto ^13^. Contudo, os resultados aqui apresentados sugerem que a distribuição dos serviços de atenção ao parto pode não estar adequada à demanda de gestantes. A institucionalização de uma rede de atenção homogênea num território heterogêneo é um grande desafio. Todas as RS do estado apresentam déficit de leitos obstétricos ^26^. A RS Baía da Ilha Grande tem alta fragmentação e dispersão das áreas ocupadas, além de ter sofrido uma série de desarticulações (demora/desabilitação de leitos, falta de apoio técnico, falta de recursos etc.) nos últimos anos. O fato de a maior porcentagem de gestantes que precisou se deslocar ser de residentes na RS Centro Sul pode ter relação com a presença de maternidades públicas em apenas 3 dos 11 municípios da RS ^26^. Apesar de o último diagnóstico regional (2017) ter constatado que a RS Metropolitana II foi autossuficiente para atender suas residentes ^26^, os resultados mostram um aumento de quase 20 pontos percentuais no deslocamento de gestantes entre os dois períodos analisados. Um cenário semelhante foi evidenciado na RS Baixada Litorânea. Uma análise mais aprofundada da infraestrutura hospitalar dos serviços de atenção ao parto e das necessidades das usuárias, pode contribuir com a avaliação da efetividade da atenção e gestão em saúde.

A diferença entre o tempo de deslocamento das gestantes por carro e ônibus evidenciou as dificuldades de mobilidade urbana no estado, agravada pela forma de ocupação e organização do espaço. A RS Metropolitana I, por exemplo, corresponde a 5,16% da área total do estado e abriga cerca de 61,50% de sua população ^4^, com altas densidades demográficas. O *Relatório Global Moovit sobre o Transporte Público*^27^ apontou o Município do Rio de Janeiro como o quarto pior do mundo em tempo médio gasto no transporte público. O cenário encontrado nas RS Baía da Ilha Grande e Noroeste reflete a baixa densidade demográfica, com regiões menos urbanizadas e maiores distâncias entre as sedes municipais.

A análise de redes sociais é utilizada na avaliação da acessibilidade aos serviços de saúde ^28^, incluindo a internação para o parto ^29^. A identificação dos municípios núcleo e dos municípios polo de atração mostrou áreas geográficas de atendimento médico nas quais há maior demanda para atenção ao parto. O fato de a maioria dos núcleos e polos estar localizada nas RS metropolitanas do Rio de Janeiro é esperado e consistente com a maior concentração de instalações médicas nessa região, além da maior densidade populacional. Embora existam RS de menor densidade populacional no estado, novos centros de atenção ao parto nas RS onde o deslocamento das gestantes foi mais expressivo (Centro Sul e Baixada Litorânea) e nas quais as gestantes percorreram as maiores distâncias e levaram mais tempo para acessar o hospital (Baía da Ilha Grande e Noroeste) melhoraria o acesso aos serviços. Estudos considerando as áreas de cobertura e a capacidade de absorção hospitalar poderiam contribuir para esta análise.

A mudança temporal nos núcleos e polos de atração evidenciou a indisponibilidade de serviços de atenção ao parto em alguns municípios. Queimados e Belford Roxo, considerados polos em 2010-2011, perderam as referências das maternidades alocadas no seu território e as gestantes residentes tiveram que ser encaminhadas ao Hospital da Mãe ^26^, em Mesquita, que passou a atender 87% de não residentes. Saquarema, que aparece como núcleo em 2018-2019, passou a receber gestantes de Araruama e Iguaba Grande que sofreram descredenciamento de seus prestadores ou não tinham leitos suficientes para atendimento da demanda ^26^. Permanece a dúvida se todos os municípios núcleos e polos de atração estão preparados para atender a população não residente. Além disso, é possível que alguns dos hospitais de referência não sejam atraentes para as gestantes por diversas razões, incluindo a disponibilidade de transporte e a confiança da população. A compreensão dos fatores que contribuem para esse fluxo crescente e desequilibrado de gestantes em direção aos núcleos e polos pode fornecer informações úteis para planejar e melhorar os serviços de atenção ao parto.

A interpretação dos resultados apresentados tem algumas limitações. O SIH pode ter registros de parto insuficientes, particularmente por conta do teto de pagamento máximo de cesarianas em relação ao total de partos por hospital implementado em 2000 ^30^. Contudo, a partir da implantação da Rede Cegonha e da revogação dessa normativa em 2017 ^31^, os registros de parto se tornaram mais confiáveis. O SIH ainda é amplamente utilizado para a formulação de indicadores para o monitoramento e planejamento estratégico de ações em saúde. Outras medidas poderiam contribuir para a caracterização do problema, como custo de viagem e peregrinação das gestantes no momento do parto, mas essas informações não estão facilmente disponíveis. Analisamos um dos muitos aspectos da rede de serviços de atenção materno-infantil: a acessibilidade geográfica ao parto hospitalar de risco habitual. Uma análise abrangente da infraestrutura hospitalar dessa rede de serviços e de sua demanda, bem como dos serviços de atenção ao parto de alto risco fornecerá informações adicionais para o planejamento e a melhoria da acessibilidade das gestantes.

## Conclusões

Os resultados deste estudo são úteis para informar os usuários e gestores dos serviços de atenção à saúde materno-infantil do Estado do Rio de Janeiro que: (1) aproximadamente 19% das gestantes precisam se deslocar de seu município de residência para realizar parto hospitalar no SUS; (2) os deslocamentos geralmente obedecem aos limites das RS; (3) na última década, houve aumento do percentual de gestantes que se deslocaram, acompanhado por um aumento das distâncias percorridas e do tempo de deslocamento para chegar ao hospital; (4) há desigualdades regionais importantes que persistiram na última década, dificultando o acesso das gestantes residentes nas RS Centro Sul, Baía da Ilha Grande e Noroeste; e (5) os núcleos e polos de atração refletem a indisponibilidade de serviços em alguns municípios e ressaltam a necessidade de ajustar o acesso à atenção ao parto. O estudo agrega valor às pesquisas sobre melhoria do acesso aos serviços de saúde, alinhado aos Objetivos de Desenvolvimento Sustentável da Organização das Nações Unidas de 2030, e fornece dados relevantes para o planejamento do acesso à atenção ao parto e nascimento no contexto da regionalização do SUS.
